# Genetically engineered T cells for cancer immunotherapy

**DOI:** 10.1038/s41392-019-0070-9

**Published:** 2019-09-20

**Authors:** Dan Li, Xue Li, Wei-Lin Zhou, Yong Huang, Xiao Liang, Lin Jiang, Xiao Yang, Jie Sun, Zonghai Li, Wei-Dong Han, Wei Wang

**Affiliations:** 1 Department of Biotherapy, State Key Laboratory of Biotherapy and Cancer Center, West China Hospital, Sichuan University, and the Collaborative Innovation Center for Biotherapy, 610041 Chengdu, China; 2 Department of Medical Oncology, Cancer Center, West China Hospital, West China Medical School, Sichuan University, and the Collaborative Innovation Center for Biotherapy, 610041 Chengdu, China; 3 Department of Cell Biology, and Bone Marrow Transplantation Center of the First Affiliated Hospital, Zhejiang University School of Medicine, 310058 Zhejiang, China; 4 Institute of Hematology, Zhejiang University & Laboratory of Stem cell and Immunotherapy Engineering, 310058 Zhejing, China; 5 State Key Laboratory of Oncogenes and Related Genes, Shanghai Cancer Institute, Renji Hospital, Shanghai Jiaotong University School of Medicine, 200032 Shanghai, China; 6 CARsgen Therapeutics, 200032 Shanghai, China; 7 Molecular & Immunological Department, Biotherapeutic Department, Chinese PLA General Hospital, No. 28 Fuxing Road, 100853 Beijing, China

**Keywords:** Molecular medicine, Drug development

## Abstract

T cells in the immune system protect the human body from infection by pathogens and clear mutant cells through specific recognition by T cell receptors (TCRs). Cancer immunotherapy, by relying on this basic recognition method, boosts the antitumor efficacy of T cells by unleashing the inhibition of immune checkpoints and expands adaptive immunity by facilitating the adoptive transfer of genetically engineered T cells. T cells genetically equipped with chimeric antigen receptors (CARs) or TCRs have shown remarkable effectiveness in treating some hematological malignancies, although the efficacy of engineered T cells in treating solid tumors is far from satisfactory. In this review, we summarize the development of genetically engineered T cells, outline the most recent studies investigating genetically engineered T cells for cancer immunotherapy, and discuss strategies for improving the performance of these T cells in fighting cancers.

## Introduction

T cells play central roles in cell-mediated adaptive immunity. Since researchers identified the molecular evidence of T cell receptors (TCRs) in the 1980s^1,2^, the recognition of antigens by TCRs has been heavily investigated, and the molecular mechanisms governing this process have been elucidated^3,4^, laying the foundation for cancer immunotherapy.

Cancer immunotherapy exploits the body’s own immune system to fight against cancer. This therapy was designated as an annual scientific breakthrough in 2013 by Science magazine and has exhibited promising antitumor efficacy in recent years^5–7^. Cancer immunotherapies are categorized as immune checkpoint inhibitors (ICIs), adoptive cell therapies (ACTs), and tumor vaccines^8,9^. Numerous patients with advanced tumors have benefited from cancer immunotherapy, and some have achieved complete remission.

ACT has long been used to treat cancers and other diseases. The adoptive transfer of T lymphocytes expanded ex vivo has shown limited antitumor efficacy, as these T lymphocytes lack specificity against tumor cells. To enhance the efficacy of ACT, the infusion of tumor-infiltrating lymphocytes (TILs) with specificity against the tumor cells in patients with preconditioning regimens substantially improved the efficacy of the treatment^10–13^. After cloning the *TCR* gene of the TIL, it is possible to endow T cells with defined specificity by transferring the cloned *TCR* gene^14,15^. T cells engineered by viral vectors to express the *TCR* gene with defined specificity have shown considerable benefit for the treatment of cancers^16,17^, although there are many limitations of TCR-engineered T (TCR-T) cells, including HLA restriction, side effects, and the lack of a sufficiently broad *TCR* gene repertoire with defined specificity^18,19^. Chimeric antigen receptor-modified T (CAR-T) cells, which are genetically engineered to express CAR molecules targeting surface antigens on tumor cells and other cells, can overcome some of the limitations of TCR-T cells^20,21^. Since the first demonstration of cytotoxicity to target-bearing cells^18,20–23^, CAR-T cells have been extensively investigated in preclinical and clinical studies and have exhibited dramatic efficacy in treating hematological malignancies^24–28^, although moderate effects have been obtained for the treatment of solid tumors^29–31^.

In this review, we summarize the recent investigations of genetically engineered T cells, mostly focusing on CAR construct optimization, clinical efficacy, and strategies to overcome resistance and other limitations, as well as the outlook for future applications of genetically engineered T cells to cancer therapy.

## Rationale for the emergence of genetically engineered T cells

T cells gain autoimmune tolerance after the positive selection of thymocytes^32^ and play pivotal roles in adaptive immunity^33^. T cells can provide protective immunity through TCR recognition of foreign antigenic peptides presented by antigen-presenting cells (APCs)^34,35^, by which T cells might combat tumor cells^35,36^. The adoptive transfer of T cells was first investigated in the treatment of localized and disseminated lymphoma, and tumors regressed after the infusion of T cells in a syngeneic mouse model^37^; subsequently, studies have investigated the clinical applications of T cells and other immune cells to fight cancers and other diseases. In fact, T cells infused for the treatment of cancers have been manipulated ex vivo by using different strategies: e.g., LAKs (lymphokine-activated killers) are T cells that proliferate after induction with interleukin (IL)-2. To enhance the specificity of transferred T cells, investigators have attempted to activate and induce proliferation in tumor-specific T cells by using dendritic cells exposed to tumor cell lysates^38–40^, although only moderate clinical benefits have been obtained in these clinical trials^41–43^. For the treatment of hematological malignancies, the transfer of allogeneic T cells is an important strategy to induce tumor elimination^44^, but it damaged normal tissue and visceral organs in recipients, resulting in graft-versus-host disease (GVHD)^45,46^. The prevention of GVHD by T cell depletion or host-specific allogenic T cell elimination has been proven to be effective and to improve long-time survival^47–49^.

Well-tested ex vivo expansion strategies^50,51^ warrant sufficient production of the isolated T cells for clinical applications, while the antitumor efficacy of adoptive transfer LAKs and cytokine-induced killer cells is moderate, mainly due to a lack of sufficient effector T cells specifically targeting tumor cells^52–54^. TILs are effector T cells that leave the blood and infiltrate into tumor tissue to attack tumor cells. TILs theoretically load TCRs specific to tumor antigens, and it has been found that TILs expanded ex vivo have an antitumor efficacy that is enhanced 50–100-fold compared with that of IL-2 alone^55^. Pioneering clinical trials initiated by Rosenberg and colleagues using expanded TILs for the treatment of melanoma and other tumors demonstrated that the adoptive transfer of autologous TILs is efficacious in regressing primary tumor cells and reducing metastasis^56^. After decades of research^43–47^, the adoptive transfer of TILs has been demonstrated to be one of the most important cancer immunotherapies for the treatment of melanoma and several other tumors^10^.

However, the many hurdles facing the use of TILs limit the antitumor capacity of TIL-based immunotherapy. TILs directly recognize antigens presented on the surface of tumor cells in the form of major histocompatibility complex (MHC)–peptide complexes^57,58^. Because tumor-associated antigen (TAA) is also expressed on self-tissue, immune tolerance occurs when using TILs exposed to p-MHCs derived from TAAs, resulting in unresponsive T cells^59^. In addition, tumor cells can escape immune surveillance for several reasons, including the downregulation of MHC molecules and autoantigens, leading to immunosuppression and weak immunogenicity^60–62^. In addition, the TILs from a considerable proportion of patients cannot be obtained or expanded enough for infusion, which promotes the engineering of T cells by transducing *TCR* genes with known specificity to tumor antigens.

TCR-T cells showed strong tumor-killing ability in murine models and mediated the regression of tumors in clinical trials^14,17,63^, demonstrating that the specificity of T cells can be redirected against the indicated antigens to mediate immunity to cancer. However, the immunity of TILs or TCR-T cell-based cancer immunotherapy is MHC-restricted, since the recognition of MHC-presented antigens by naive or transduced TCRs is the underlying molecular mechanism. In the 1980s, investigators pioneered a method of redirecting the specificity of T cells by introducing genes encoding artificial TCR-like molecules composed of single-chain variable fragments (scFv) of antibodies, spacers, transmembrane domains, and intracellular domains^20,21^, known as CARs. Unlike TCR, CAR recognizes surface antigens on target cells via the scFv, and CAR-T cells thus elicit cytotoxicity towards target cells in an MHC-independent manner, which broadens the application of genetically engineered T cells. Many gene transfer tools have been exploited for the engineering of T cells, including retroviral vectors^64^, liposomes^65^, electroporation^66^, and other gene-editing strategies^67–69^. The safety and antitumor capacity of gene-engineered T cells have been demonstrated^31,70–72^, making genetically modified T cell-based cancer immunotherapy a promising treatment regimen for hematological and solid tumors.

## Genetically engineered T cells for treating hematological malignancies

### Use of CAR-T cells for treating hematological malignancies

After the development of CARs, CAR-T cell-based immunotherapy was utilized in the treatment of lymphoma, leukemia, myeloma, and other hematological malignancies (Table 1)^21,73^. Currently, it is well accepted that CAR-T therapy is efficacious in the treatment of hematological malignancies and exhibits controllable and tolerable toxicity.

**Table 1:** Application of engineered T cells in clinical trials for treating hematological malignancies

Type of the arm	Target/construct	Phase	No. of patients/disease	Efficacy	Reference
CAR-T therapy					
Tisagenlecleucel, CTL019	CD19-(4-1BB)-(CD3-zeta)	Phase II	75, RR-ALL children and young adults	OR 81% (3 months) OS 76% (12 months)	81
Tisagenlecleucel, CTL019	CD19-(4-1BB)-(CD3-zeta)	Phase II	17, R/R CLL	ORR, 53%; CR, 35%	82
Tisagenlecleucel, CTL019	CD19-(4-1BB)-(CD3-zeta)	Case series	14, FL	ORR, 79%; CR, 71%	83
Tisagenlecleucel, CTL019	CD19-(4-1BB)-(CD3-zeta)	Phase I after ASCT	10, MM	CRS, 10%; longer progression-free survival 20%	84
JCAR017	CD19-(4-1BB)-(CD3-zeta)	Phase II	68, R/R DLBCL	ORR 75%; CRR 37%	85
Axicabtagene ciloleuce, KTE-C19	CD19-(CD28)-(CD3-zeta)	Phase II	111, R/R DLBCL	ORR, 82%; CR, 40%	26
CD20 CAR-T	CD19-(CD3-zeta)	Phase I	7, FL and MCL	PR, 14.2%; CR, 28.5%	16
CD20 CAR-T	CD20-(4-1BB)-(CD3-zeta)	Phase II	11, R/R NHL, primarily DLBCL	ORR 82%; CRR 55%	95
CD22-CAR T	CD22-(4-1BB)-(CD3-zeta)	Phase I	21, RR-ALL children and young adults	ORR, 53%	90
bb2121	BCMA CAR-T	Phase I	20, R/R-MM	ORR 89%; RR 100%	106
LCAR-B38M	BCMA CAR-T	Phase I	19, R/R-MM	ORR 100%; 32% MRD-negative CR, and 32% nCR	84
κ or λ light chain	κ-directed CAR	Phase I	9, NHL/CLL	PR 11%	118
TCR-T therapy					
NY-ESO-1-LAGE-1	Antigens NY-ESO-1 and LAGE-	Phase I/II (with ASCT)	20, MM	70% CR or nCR	70
WT1 TCR-T	Antigen WT1	Phase I/II (with)	12, AML	66% CR	387
Bispecific antibodies					
Blinatumomab	CD19-CD3	Phase II	21, RR-DLBCL	ORR 43%; CRR 19%	148
Natural killer cell therapy					
CAR-NK	Cd19-(NK-92)	Registered clinical trials	CD19-positive B cell malignancies	Unpublished	163
CAR-NK	Cd33-(NK-92)	Registered clinical trials	AML	Unpublished	163
CAR-NK	Cd7-(NK-92)	Registered clinical trials	CD7-positive leukemia or lymphoma	Unpublished	163
CAR-NK	CD19-(cord blood)	Registered clinical trials	CD19-positive leukemia or lymphoma	Unpublished	163

Among the CAR-T cells used for treating hematological cancers, CD19 CAR-T cells are the most prevalent, and hundreds of clinical trials using CD19 CAR-T cells are underway. CD19 CAR-T cells, which target conservatively and extensively expressed CD19 in B-cell lymphomas or leukemias^74–76^, have shown promising outcomes in several trials for treating relapsed, refractory B cell (R/R) cancers^28,77–79^, leading the U.S. Food and Drug Administration (FDA) to approve the first CAR-T cell product, tisagenlecleucel (Kymriah or CTL019), for the treatment of acute lymphoblastic leukemia (ALL) in children and adults^80^. The mostly recently updated data showed that, in a trial of CD19 CAR-T therapy in which 75 evaluable patients participated in the study, 81% of them achieved 3-month overall remission and 76% achieved 12-month overall survival (OS; the median OS was 19 months)^81^. Furthermore, CTL019 has been tested in R/R chronic lymphocytic leukemia (CLL; 17 evaluable patients; overall response rate (ORR) was 53%, complete response (CR) rate was 35%)^82^, follicular lymphoma (14 evaluable patients, ORR was 79%, CR was 71% after 6 months)^83^, multiple myeloma (MM) (10 patients, 1 with cytokine release syndrome (CRS) and 2 with longer progression-free survival)^84^. In addition to CTL019, another product, known as axicabtagene ciloleucel, exhibited robust efficacy in treating R/R diffuse large B cell lymphoma (DLBCL). Phase II trials demonstrated an ORR of 82%, with a CR rate of 40% at a median follow-up of 15.2 months^26^. JCAR017, a well-regarded product produced by Juno Therapeutics, resulted in a dramatic ORR of 75% and a 6-month CR of 37% in 68 R/R DLBCL patients^85^. Overall, CD19 CAR-T therapy showed encouraging therapeutic effects and safety, illustrating that CD19 CAR-T has advantages for the treatment of hematological malignancies, especially B cell malignancies^84^. Despite the curative efficacies of CD19 CAR-T cells, their side effects, resulting in normal B cell dysplasia, lethal CRS, and neurotoxicity, warrant further study^79,84,86,87^. In addition, relapse after CR post-CD19 CAR-T infusion is another concern for users of this therapy. Several mechanisms have been uncovered that were involved in relapse, including CD19 loss, CD19 antigen masking, and trogocytosis^88,89^. CD22, an alternative surface marker expressed by B cell leukemia and lymphoma cells, can be targeted by CAR-T therapy. The sequential infusion of CD22 CAR-T cells post-CD19 CAR-T therapy can lead to the remission of cancer that relapsed after CD19 CAR-T therapy^90^. In fact, previous studies demonstrated the antitumor capacity of CD22 CAR-T therapy^91,92^, and earlier clinical studies of CD22 CAR-T cells in ALL were published in 2018^90^. Compared with CD19 CAR-T cells, CD22 CAR-T cells showed comparable antileukemia cytotoxicity^90^. Meanwhile, there is no evidence for neurological toxicity and seizures resulting from CD22 CAR-T therapy, which has been observed in CD19 CAR-T therapy^90,93^. To maximize the benefits of CD19 and CD22 CAR-T therapy for hematological malignancies, the sequential or simultaneous combination of the two different CAR-T products should be further studied.

CD20 is a classic target for lymphoma treatment. The targeting of CD20 by rituximab is efficacious for the treatment of non-Hodgkin’s lymphoma (NHL). Efforts have been made to use CAR-T cells targeting CD20 to treat lymphoma. Although the level of CD20 is not comparable to that of CD19, CD20 is also frequently expressed in lymphoid malignancies and B cell acute lymphocytic leukemia (B-ALL)^91,92,94^. CD20 CAR-T cells have been evaluated for efficacy and safety, and preclinical investigations have demonstrated similar anti-lymphoma activity compared to CD22 CAR-T cell therapy^91^. In a clinical trial of seven patients with follicular or mantle cell lymphomas treated with first-generation CD20 CAR-T therapy, three patients showed a positive response (one partial response (PR), two CR)^16^. In a clinical trial for the treatment of R/R NHL, primarily DLBCL, >80% of patients showed an objective response^95^. These studies have proven that CD20 CAR-T therapy is efficacious with minimal toxicity, but its poor persistence might be an obstacle to sustained antitumor efficacy for first-generation CAR-T cells. Second-generation CD20 CAR-T cells may lead to improved persistence by adding a costimulatory domain (such as 4-1BB, CD28 or a dual costimulatory molecule)^96^. Clinical data for second-generation CAR-T cells targeting CD20 have shown their robust antitumor capacity and minimal toxicity for the treatment of CD20-expressing lymphoma^96–98^. In leukemia and lymphoma, CD19, CD22, and CD20 have different expressional hierarchies in tumor cells. However, a head-to-head comparison of the antitumor efficacy of CAR-T cells targeting different antigens is needed to determine whether the selection of the antigen affects the clinical efficacy of CAR-T cells.

MM is another hematological malignancy that is derived from plasma cells. MM is one of the most studied hematological cancers that CAR-T therapy targets. MM cells were found to express ultralow levels of CD19^24,99^, and CD19 CAR-T therapy for treating MM resulted in a durable CR >1 year, despite the absence of CD19 expression post-CD19 CAR-T cell infusion^24^. B cell mature antigen (BCMA) belongs to the tumor necrosis factor receptor family^100–102^ and is specifically expressed on MM cells, plasma cells, and partial memory B cells^100,103–105^, making BCMA an ideal marker for targeting by CAR-T cells. Two BCMA CAR-T cell products (bb2121 and LCAR-B38M) were subject to clinical trials for the treatment of MM. The data from the bb2121 phase I dose-escalation trial showed that the CR rate reached 71% among the 21 patients treated^106^. LCAR-B38M treatment in 57 recruited patients produced an ORR of 88% and a CR of 74%^107^. These initial efforts using BCMA CAR-T cells for MM treatment indicated that more evidence of safety and efficacy is needed. Other targets, such as CD38, CS-1, and CD138, are targeted by CAR-T cells for the treatment of MM. Monoclonal antibodies against CD38 and CS-1 have been approved by the FDA for the treatment of MM. Indatuximab ravtansine, a monoclonal antibody (mAb) against CD138 conjugated with maytansinoid, has been developed for MM treatment. However, CAR-T cells recognizing MM targets need to be meticulously manipulated, especially because of the side effects caused by “on-target, off-tumor” toxicity^108–111^.

Receptor tyrosine kinase-like orphan receptor 1 (ROR1) is abnormally expressed on malignant B cells and at lower levels on normal cells and tissue^110,112–115^. Although CAR-T cells recognizing ROR1 elicited strong cytotoxicity in tumor cells, toxicity in normal cells expressing low levels of ROR1 was detected^92,112,116^, and further evidence of safety and efficacy are needed prior to the application of ROR1 CAR-T cells. In human B-cells, the immunoglobulin-κ (Ig κ) and Ig λ light chains are expressed at a certain ratio, which ranges from 4:1 to 0.5:1. Light-chain CAR-T cells can recognize cells histologically and do not target 20–80% of normal B cells and plasma cells^110,117^. Ten NHL/MM/CLL patients were treated with κ-redirected CAR-T cells in a clinical trial. Among the five patients with relapsed NHL, two achieved complete remission (after two and three infusions), one had a PR, and two progressed. Three MM patients showed stable disease, and the two CLL patients progressed before or shortly after the 6-week evaluation^118^. CD30, a biomarker targeted by the brentuximab vedotin mAb, is expressed on all Hodgkin’s lymphomas (HLs) and a portion of NHLs. CAR-T cells recognizing CD30 showed antitumor capability in preclinical and clinical studies^86,119,120^. CAR-T cells specifically recognizing other targets, including CD123 (the IL-3 receptor α chain)^121–123^, CD33^124,125^, CD44v6, and Lewis Y^126^ antigen, were subjected to preclinical and clinical investigation.

### Use of TCR-engineered T (TCR-T) cells for treating hematological malignancies

The application of TCR-T cells as a novel adoptive immunotherapy^127,128^ has shown encouraging results in the treatment of several advanced cancers^127,129^. TCR-T cells were able to detect intracellular antigens more sensitively via the MHC system^130^. Clinical investigations using TCR-T cells for the treatment of metastatic melanoma, synovial sarcoma, and colorectal cancer have achieved significant success^17,70,131–134^. TCR-T cells recognizing New York esophageal squamous cell carcinoma (NY-ESO-1 or CTAG1A) have been used to treat patients with advanced MM and have resulted in durable CR^135^. In a clinical trial, 80% of patients with MM (20 in total) who received NY-ESO-1 TCR-T cell therapy achieved objective responses without severe toxicity. Although TCR-T cell therapy presents new therapeutic opportunities for MM patients and other tumor patients^70,136–138^, safety should be of primary concern for other targets^139,140^, especially when the TCRs used for antigen recognition are modified^141^.

### Bispecific antibodies for treating hematological malignancies

Bispecific T cell engagers (BiTE) are engineered mAbs consisting of two scFvs with affinity for CD3 and tumor antigens^142^. BiTE shares many similarities with CAR-T cells and exhibits remarkable efficacy as a cancer therapy. The promising efficacy of BiTEs in the treatment of hematological malignancies has been demonstrated in clinical trials^143,144^. Blinatumomab (or MT103), which is specific for CD3 and CD19 and was the first BiTE approved by the FDA, has been used in several trials to treat lymphoma, ALL, and MM^145–147^. Its promising performance, as shown by the clinical data (especially in R/R B-ALL), has made it known as an “off-the-shelf” product that is easy to utilize compared with CD19 CAR-T cells^148^. However, adverse events usually accompany the administration of blinatumomab. The most common side effects are neurotoxicity and CRS^149–154^. Moreover, the half-life of BiTE is shorter, resulting in a more complicated treatment process. CD20/CD3 BiTEs (TBTA05 and CD20Bi) have been evaluated in clinical trials^145^. CD3/BCMA and CD3/CD38 BiTEs have completed preclinical evaluation^103,155,156^. CD3 linked with CD123 and CD33 BiTEs has been evaluated in clinical trials for AML patients^145,157^. In the near future, BiTEs targeting different antigens expressed by hematological malignancies may provide alternatives for patients, and the comparison of BiTE with CAR-T cells in the treatment of the same targets will improve our understanding of the treatment of cancer by immunotherapy.

### Use of genetically engineered natural killer (NK) cells for treating hematological malignancies

NK cells are mainly derived from bone marrow CD34 lymphocytes^158,159^. NK cells recognize and elicit rapid responses against virus-infected cells and tumor cells and serve as important innate effector cells^160–162^. NK cell-based cancer immunotherapy, including the adoptive transfer of NK cells combined with NK cells and mAbs (such as ICIs), can be used to induce antibody-specific cytotoxicity, and genetically engineered NK cells can be used to induce antitumor effects. Compared with CAR-T cells, CAR-NK cells mainly produce interferon (IFN)-γ and granulocyte macrophages colony-stimulating factor (GM-CSF), thereby reducing the potential CRS^163,164^. NK cells have a shorter survival time than CAR-T cells, and the incidence of side effects is further reduced, although it is likely that the antitumor efficacy is decreased^165^.

Natural killer T cells (NKTs) are a group of special T cell subsets that express both T cell surface markers and NK cell surface markers. Although NKTs in the body account for only 1/1000 of the total number of immune cells, the antitumor activity of NKTs is robust, but the reason for this is not fully understood. Preclinical data suggest that NKTs express the surface markers of both NK cells and CD8 T cells and produce more cell killing enzymes that kill tumor cells^166^. Evidence of the role of NKT cells in tumor immune surveillance and the antitumor potential of ligand-activated NKT cells have been demonstrated in a mouse model, increasing the enthusiasm for conducting further antitumor potential investigations^161,167,168^.

Studies have investigated the engineering of NKT cells to improve their antitumor potential. Hecezy et al. demonstrated that NKT cells modified by CARs with specificity for GD2 showed specific toxicity against GD2-positive tumor cells, increases in in vivo survival, and potent antitumor activity in a mouse model of a solid tumor^163^. GD2 CAR-NKT cell therapy is now being tested in clinical trials to determine its safety and efficacy for treating neuroblastoma (NCT03294954). NKT cells were also engineered for treating lymphoma. Tian et al. documented that CD62L is crucial for the antitumor activity of CAR-NKT cells^169^. CD62L is the receptor of sulfated sialyl Lewis X antigen and functions in peripheral lymph node homing. It has been demonstrated that CD62L-positive but not CD62L-negative CD19 CAR-NKT cells exhibit prolonged in vivo persistence and superior anti-lymphoma activity^169^. It is hoped that CD62L-positive CD19 CAR-NKT cells can be tested for safety and antitumor potential to provide alternative engineered immune cells for treating hematological malignancies. All of these therapies are summarized in Table 1.

## Use of genetically engineered T cells for treating solid tumors

Genetically engineered T cells have long been employed to treat solid tumors^17,30,170^. The clinical efficacy of this type of treatment is far from satisfactory compared with that achieved in treating hematological malignancies^24,171–173^. Considerable research has been conducted in an attempt to enhance the antitumor activities of CAR-T and TCR-T cells, and different strategies aiming to determine the efficacy and safety of CAR-T therapy are being tested in clinical trials for the treatment of cancers, such as breast cancer, sarcoma, and neuroblastoma (Table 2). The first clinical application of CAR-T therapy for cancer treatment was the use of CAR-T cells recognizing carbonic anhydrase IX (CAIX) for the treatment of metastatic renal cell carcinoma, which showed moderate antitumor activity^30^. Other results of clinical trials that used genetically engineered T cells to treat solid tumors have been barely comparable to those achieved with CAR-T therapy for leukemia and lymphoma^174^.

**Table 2:** Published clinical studies using CAR-T cells for treating solid tumors

Targeted antigen	Disease	Vector	CAR generation	Sponsor	NCT identifier	Reference
FRa	Ovarian cancer	Retrovirus	First	National Cancer Institute	NA	29
Mesothelin	Pancreatic cancer	mRNA	Second	University of Pennsylvania	NA	388
c-MET	Breast cancer	mRNA	Second	University of Pennsylvania	NCT01837602	389
EGFRvIII	Glioblastoma	Lentiviral	Second	University of Pennsylvania	NCT02209376	390
CEACAM5	CRC	Retrovirus	First	The University of Manchester	NCT01212887	391
CEA	CRC	Lentivirus	Second	Third Military Medical University	NCT02349724	175
HER2	Glioblastoma		Second	Baylor College of Medicine	NCT01109095	189
GD2	Neuroblastoma	Retrovirus	First	Baylor College of Medicine	NCT00085930	392
CD133	HCC, CRC, pancreatic cancer	Lentivirus	Second	Chinese PLA General Hospital	NCT02541370	393–395

*NA* not available

The most commonly used targets for CAR-T therapy are surface antigens, such as carcinoembryonic antigen (CEA) for colorectal adenocarcinoma^175,176^, fibroblast activation protein for malignant pleural mesothelioma^177^, diganglioside GD2 for neuroblastoma^178^, glioblastoma^179^, melanoma^180^, and osteosarcoma^181^, human epidermal growth factor receptor 2 (HER2) for HER2-positive sarcoma^182^, mesothelin for pancreatic cancer^183^, IL-13 receptor α (IL-13Rα) for glioma^184^, and mutant αvβ6 integrin for pancreatic tumors^185^. TCR-engineered T cells always target the p-HLA complex. In addition, the p-HLA complex can also be recognized by CAR-T cells whose scFv for binding was derived from a TCR-like antibody^186^. For these clinical trials, some outcomes have been published. Pule et al. generated EBV-specific T cells to recognize GD2 and infused these GD2 CAR-T cells into patients to treat neuroblastoma^187^. It was found that virus-specific GD2 CAR-T cells persisted and exhibited moderate anti-neuroblastoma activity. HER2 is thought to be an ideal target for cancer therapy, and many strategies have targeted HER2 to successfully treat breast cancer, gastric cancer, and other tumors. In 2010, Morgan et al. reported that the infusion of HER2 CAR-T cells to treat metastatic colon cancer caused severe adverse effects, likely due to the large number of third-generation CAR-T cells used and “on-target, off-tumor” toxicity^188^. The meticulous redesign of the clinical strategies used, including the splitting of the HER2 CAR-T cells infusion, the use of a second-generation CAR construct with different scFvs, and a reduction in the total number of CAR-T cells, effectively improved safety while maintaining antitumor efficacy^182,189^. Epidermal growth factor receptor (EGFR) is widely expressed in normal epithelial tissue, but a mutant EGFR variant, which is a biomarker of lung cancer and breast cancer, can be targeted by CAR-T cells. It was reported that EGFR CAR-T cells used for treating 11 non-small cell lung cancer patients showed efficacy in 2 patients with PRs and 5 with stable disease^190^. CEA CAR-T cells used for liver cancer treatment also showed moderate antitumor efficacy with minimal toxicity^191^.

**Fig. 1:**
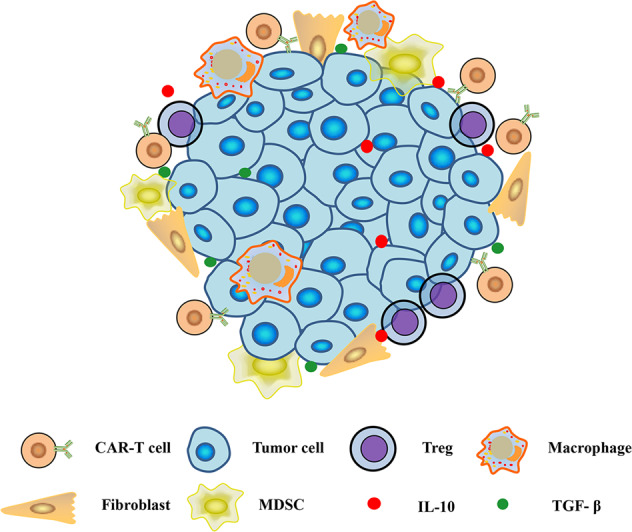
Immunosuppressive microenvironment in solid tumors. MDSC myeloid-derived suppressor cell, Treg regulatory T cell, TAM tumor-associated macrophage, TAF tumor-associated fibroblast

The reason for the moderate clinical efficacy of earlier CAR-T cells used for the treatment of solid tumors is multifactorial. Unlike hematological malignancies, solid tumors present many obstacles to CAR-T cells resulting from short-term persistence or insufficient infiltration. As a fundamental prerequisite for therapeutic efficacy, CAR-T cells need to be trafficked to the tumor lesion. Once they accumulate in the vicinity, they must efficiently infiltrate into the tumor. After migrating into the solid tumor lesion, CAR-T cells must overcome hostile immunosuppressive elements to elicit specific cytotoxicity^192^, as shown in Fig. 1.

### T cell trafficking to tumor sites

Efforts to enhance CAR-T cell trafficking have been made. Investigators have modified CAR T cells with chemokine receptors that specifically bind chemokines produced by tumors, aiming to improve the homing of CAR-T cells to tumor sites. It has been demonstrated that enhanced CCR2b expression in mesothelin CAR-T cells and GD2 CAR-T cells leads to improved antitumor effects in malignant pleural mesothelioma and neuroblastoma due to the increased migration of CAR-T cells to tumor lesions^193,194^. In addition to CCR2 expression, the transgenic coexpression of CCR4 improved the homing of CD30 CAR-T cells to CD30+ HL that secreted CCL17 (the ligand for CCR4) and thereby improved the anti-lymphoma effects^195^.

Intravenous infusion is currently considered the standard method for adoptive cell therapy. Therefore, CAR-T cells must migrate to the area where solid tumors are present. One strategy to improve T cell trafficking is the local administration of CAR-T cells to tumor sites, including intratumoral (NCT02587689), intracranial (NCT00730613), pleural (NCT02414269), and hepatic artery (NCT01373047) delivery. In a preclinical model, Adusumillii et al.^196^ evaluated two routes of administration for mesothelin-targeted T cells using M28z CARs and demonstrated that the intrapleurally administration of CAR-T cells required 30-fold fewer M28z T cells to induce long-term complete remission compared to systemically infused CAR-T cells. Intrapleural administration resulted in enhanced antitumor efficacy and functional T cell persistence for 200 days, while intravenously delivered CAR-T cells did not achieve comparable activation, tumor eradication, or persistence^196^. The central nervous system is an immune-privileged site secondary to the blood–brain barrier (BBB). It is difficult for intravenously infused CAR-T cells to traffic to brain tumors. The local delivery of T cells via methods such as intracranial infusion could circumvent the BBB and improve the immunotherapy of brain tumors^197^. Preclinical and clinical studies have confirmed the efficacy of locally delivered CAR-T cells in treating primary and metastatic brain cancers^31,198–200^.

### Immunosuppressive microenvironment

In solid tumors, CAR-T cells face a hostile tumor microenvironment (TME), even though CAR-T cells are able to migrate to tumor sites. Solid tumors are usually infiltrated by an abundance of immune-suppressor cells, including M2 tumor-associated macrophages, myeloid-derived suppressor cells (MDSCs), and regulatory T cells (Tregs) and B cells, which protect malignant cells from the antitumor activity of the immune system^201–203^. In addition, immunosuppressive cytokines and inhibitory immune checkpoints play a crucial role in tumor pathogenesis and metastasis and limit the therapeutic potential of cancer immunotherapies^204,205^.

Preclinical investigations have demonstrated that the incorporation of costimulatory molecules, such as CD28, into CARs may help engineered CAR-T cells overcome the immunosuppressive TME mediated by Treg cells^206–208^. It has been recently reported that engineered CAR-T cells with inducible IL-18 tip the balance within the immune cell landscape toward the Th1 acute-phase response, thereby reducing the number of immunosuppressive cells, such as Tregs and CD206+ macrophage cells, and resulting in an augmented immune attack against large established tumors. Notably, this effect was achieved in fully immune-competent mice with advanced pancreatic adenocarcinoma^209^. Depleting MDSCs can also boost T cell responses. A study showed that the administration of GD2 CAR-T cells in combination with MDSC depletion led to significant antitumor efficacy in a xenograft sarcoma model, while CAR-T cells alone elicited minimal antitumor activity^210^. In addition, Noman et al.^211^ demonstrated that programmed death ligand 1 (PD-L1) blockade prevented T cell suppression by MDSCs. It was also reported that the blockade of PD-L1+ MDSCs and Tregs in the TME enhanced CEA CAR-T cell antitumor capability^212^. Overall, the findings of these studies indicated that immunosuppression was mediated by suppressor cells and supported the rationale of cell preconditioning to enhance the antitumor activity of CAR-T cells.

Various immunosuppressive cytokines, such as transforming growth factor (TGF)-β and IL-10, are involved in the inhibition of engineered T cell-based cancer immunotherapy^203,213,214^. In a recent study, investigators engineered prostate-specific membrane antigen CAR-T cells to coexpress a dominant-negative TGF-β receptor and observed increased proliferation, enhanced cytokine secretion, resistance to exhaustion, long-term in vivo persistence, and the induction of tumor eradication by CAR-T cells in aggressive human prostate cancer^215^. Then a phase I clinical trial was initiated to assess CAR-T cells coexpressing TGF-βRII in patients with relapsed and refractory metastatic prostate cancer (NCT03089203). In addition, engineered CAR-T cells with the enhanced secretion of cytokines, known as TRUCKs (T cells redirected for universal cytokine killing), have been investigated^216–218^. CAR-T cells targeting MUC16 with IL-12 secretion have eradicated ovarian cancers in a preclinical trial. Engineered CAR-T cells with autocrine IL-12 expression exhibited improved antitumor activity by promoting CD8+ T cell function and potentially circumventing the inhibitory TME^219^. MUC16 TRUCKs are currently being evaluated in a clinical trial for treating solid tumors (NCT02498912)^219^. The local delivery of the chemokine RANTES (regulated and normal T cell expressed and secreted) and the cytokine IL-15 by an oncolytic virus strikingly increased the persistence of anti-GD2 CAR-T cells and enhanced the survival of established neuroblastoma cells mice^220^. We generated CAR-T cells specific for human vascular endothelial growth factor receptor 1 (VEGFR-1) and T cells that genetically expressed IL-15, and the VEGFR-1-specific CAR-T cells delayed tumor growth and formation and suppressed pulmonary metastasis in a xenograft tumor model^221^. Indeed, numerous studies have demonstrated that γ-chain (γc) family cytokines could be used to enhance the immunity of CAR-T cells. IL-2, IL-4, IL-7, IL-15, and IL-21 have been shown to mitigate the effects of immunosuppressive factors in the TME and produced remarkable enhancement of CAR-T efficacy^221–229^.

T cells have also been demonstrated to express coinhibitory receptors, termed markers of T cell exhaustion, to reduce the antitumor activities of CAR-T cells. In recent years, PD-1 has been studied as a potential target to promote CAR-T cell efficacy^230^. Strategies to manipulate PD-1 expression on CAR-T cells include the coadministration of a PD-1/PD-L1 blocking antibody, the genetic removal of PD-1 from T cells and the secretion of autocrine molecules to induce PD-1/PD-L1 blockade by CAR-T cells. Cherkassky et al. demonstrated that PD-1/PD-L1 blockade restored the antitumor activity of CD28 CAR-T cells in an orthotopic pleural mesothelioma mouse model, mechanistically confirming the role of the PD-1/PD-L1 axis in CAR-T cell exhaustion^231^. Ren et al. genetically depleted PD-1 in prostate stem cell antigen (PSCA) CAR-T cells by using the CRISPR/Cas9 gene-editing system and demonstrated their enhanced antitumor efficacy, both in vitro and in vivo, in a murine prostate cancer model^232^. Furthermore, several clinical trials of CAR-T cells expressing PD-1 antibody for the treatment of solid tumors are underway (NCT03030001, NCT02873390, NCT03179007, NCT03182816, NCT03182803, NCT03615313). Additionally, a clinical trial (NCT03399448) sponsored by University of Pennsylvania is evaluating the antitumor efficacy of NY-ESO-1-specific TCR-T cells with the elimination of PD-1 and endogenous TCR by the CRISPR/Cas9 gene-editing system for the treatment of MM, melanoma, and other tumors. It should be noted that systemic checkpoint blockade diseases, such as pneumonitis, colitis, hepatitis, and other adverse events, have occurred in many patients after PD-1 blockade treatment. In a PD-1 monotherapy clinical trial, Suzanne et al. observed grade 3 or 4 drug-related adverse events in 14% of patients, and 3 patients died from pulmonary toxicity out of a total of 296 patients^233^. Therefore, side effects should be considered when combining CAR-T cells with PD-1/PD-L1 blockade therapy. However, when PD-1 blockade was combined with CAR-T cells, no damage was observed in normal tissues, while the enhancement of the anti-breast cancer activity of CAR-T cells was maintained^234^. CAR-T cells secreting anti-PD-1 ScFv also produced minimal side effects^235^. The reduced side effects might be ascribed to the restricted secretion of PD-1 blockade antibodies in areas of CAR-T distribution or the selective elimination of PD-1 specifically on CAR-T cells. It is expected that the combination of CAR-T therapy with anti-PD-1 therapy will increase the efficacy of cancer immunotherapy.

## Evolution of genetically engineered T cells

The first clinical application of CAR-T cells was for the treatment of metastatic renal cell carcinoma by the infusion of CAR-T cells recognizing CAIX^236^. CAIX CAR-T cells successfully targeted CAIX-expressing tumor cells but also recognized CAIX-expressing normal tissues, resulting in so-called “on-target, off-tumor” toxicity and grade 2-4 enzyme disturbances^236^. The antitumor efficacy of CAIX CAR-T was moderate, mainly due to the construction of a CAR with an intracellular CD3ζ motif, which failed to induce robust in vivo antitumor effects after engaging with tumor cells^236,237^. To obtain ideal antitumor efficacy and prevent severe adverse effects, the CAR construct was optimized. The CAR molecule consists of four parts: the extracellular scFv, the transmembrane domain, the costimulatory domain and the CD3ζ signal domain, which are intracellular^238^.

### Binding domain

Each part of the CAR construct can be modified to improve the biological function of CAR-T cells. The scFv or other molecule used for the binding domain exerts pivotal effects on the targeting function of CAR-T cells and can be further optimized^239,240^. This approach may employ human scFvs for CAR construct design to improve the long-term survival of CAR-T cells, whereas a potential human anti-mouse antibody reaction may eliminate CAR-T cells that use a murine scFv for binding and lead to poor persistence. In recent years, efforts have been made to replace scFvs with nanobodies derived from camelids^241^, and preclinical studies have shown that nanobody-based CAR-T cells showed improved flexibility in antigen recognition and achieved comparable efficacy when treating different tumors^242,243^. CAR-T cells attack cells expressing the CAR-recognized antigens, which are always TAAs. TAAs are also expressed on normal tissues at lower levels compared with those of tumors. To maximally mitigate “on-target, off-tumor” toxicity, the affinity of the scFv in the CAR construct is meticulously tuned to selectively recognize tumor cells having higher levels of TAAs while sparing normal tissues with low levels of antigen^244,245^. CAR-T cells always recognize the antigens on the surfaces of the target cells. The TCR-like antibody specifically binds the p-HLA resembling the TCR, since it binds neither the presented peptide nor the HLA molecule alone. CAR-T cells that use TCR-like scFvs for binding can recognize intracellular antigens^246^. CAR-T cells with TCR-like scFvs recognizing alpha fetoprotein have shown robust tumor-inhibiting effects in the treatment of liver cancer^186^. Indeed, some other molecules have been used for binding, e.g., ligands or cytokines binding to a specific receptor, which are antigens that the targeted cells can recognize^247,248^.

Antigens for TCR-T cells have been derived from melanoma antigens, such as GP100, MART1^249^, NY-ESO-1^250^, MAGE family members^251^, and WT1^252^, and the clinical application of TCR-T cell therapy has achieved substantial efficacy in the treatment of melanoma^17^. Similar to CAR-T therapy, the TME often inhibits the efficacy of TCR-T cell therapy. Preconditioning strategies, including lymphodepletion by nonmyeloablative chemotherapy or intensive myeloablative chemoradiotherapy, have been demonstrated to improve the antitumor efficacy of TILs^253^. Preconditioning chemotherapy with cyclophosphamide plus fludarabine has shown promise in improving the efficacy of TCR-T and CAR-T cell therapy^26,83^. The affinity of TCRs are significantly associated with TCR-T cell efficacy. Affinity optimization produced improved tumor-killing ability ex vivo. However, the modification of affinity must be meticulously implemented, since improper modification can lead to cross-reactions and result in lethal adverse effects^141,254,255^.

### Intracellular domain

Distinct from the extracellular binding domain, the intracellular domain of CAR results in signaling transduction initiated by the binding of the extracellular domain to the antigen. Recently, the evolution of CAR-T cells has focused on the costimulatory intracellular domain and resulted in the substantial enhancement of antitumor efficacy. First-generation CAR-T cells, which contain a CAR construct with only CD3ζ in the intracellular domain, showed limited antitumor efficacy in clinical trials^16,256^, mainly due to the lack of costimulatory molecules that are essential for T cell responses to antigens^257,258^. In fact, the intracellular signal transduction domain always includes the indispensable CD3ζ chain, which contains immunoreceptor tyrosine-based activation motifs (ITAMs) that function in signal transduction^259^, and one or more intracellular costimulatory molecules, such as CD28, 4-1BB (CD137), or CD27, to transmit activation signals^180,260–270^. In terms of the intracellular signal domain, CARs have evolved from the first to the fourth generation. The intracellular signal transduction domain of first-generation CARs consisted of ITAMs from the CD3ζ chain of the TCR. It has been demonstrated that first generation of CAR-T cells persist in the recipient without long-term survival and robust proliferation^29,256^. Then costimulatory signal molecules, including CD28, CD137, CD27, and OX40, were integrated into CAR constructs to enhance the persistence, proliferation, and cytokine secretion of T cells^271–274^. Second-generation CAR-T cells, bearing one costimulatory domain and CD3ζ, have achieved remarkable clinical efficacy in treating B-ALL^275–277^, although the optimal costimulatory molecules for signaling transduction in CAR constructs remain to be determined. The third-generation CAR construct includes CD3ζ and two costimulatory molecules from among CD28, 4-1BB, and OX40, further improving the proliferation and survival of CAR-T cells after infusion^8,91,278,279^. Fourth-generation CAR-T cells are TRUCKs, and TRUCKs contain CAR molecules and a CAR-inducible cytokine (IL-12), which can deposit cytokines in the targeted tumor area and recruit a second wave of immune cells, such as macrophages and NK cells^217,280,281^. Currently, second- and third-generation CAR-T cells are mainly used for clinical applications.

**Fig. 2:**
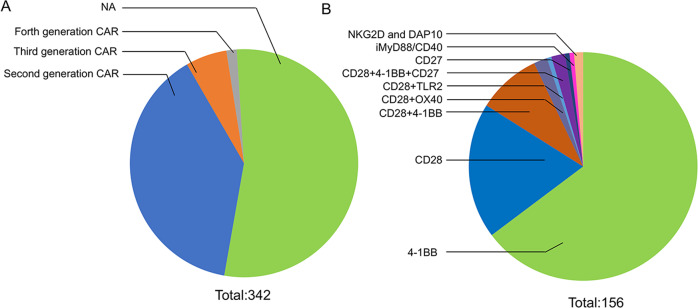
Intracellular costimulatory domain of a CAR construct used in CAR-T cells tested in clinical trials. The data were obtained from https://clinicaltrials.gov, which was accessed on January 30, 2019. **a** Diagram of clinical trials of CAR-T cells from different generations. There are 342 registered trials categorized as CAR-T cell trials (second generation: 133, third generation: 20, fourth generation: 5, NA, not available). **b** Diagram of clinical trials of CAR-T cells using different costimulatory molecules. A total of 156 available CAR constructs with different costimulatory molecules were specifically indicated, and a pie diagram is presented that shows the percentages of trials using cells with different costimulatory domains (4-1BB: 64.7%, CD28: 19.2%, CD28+4-1BB: 9%, CD28+OX40: 2%, CD28+TLR2: 0.6%, CD28+4-1BB+CD27: 2%, CD27: 0.6%, iMyD88/CD40: 0.6%, NKG2D and DAP10: 1.2%)

Although many intracellular domains in costimulatory molecules can improve the tumor-killing activities of CAR-T cells, CD28 and 4-1BB intracellular domains are the most commonly used costimulatory domains, and CAR-T cells with either CD28 or the 4-1BB intracellular domain have shown dramatic antitumor capability and tolerable toxicity in clinical trials^28,271,272,275^. When analyzing the mostly recently updated CAR-T clinical trials (342 in total; 156 CAR constructs are available) worldwide at https://clinicaltrials.gov, it was found that most CAR constructs used in the CAR-T clinical trials are second-generation CAR constructs. Twenty clinical trials are using the third-generation CAR construct, and five are using fourth-generation CAR constructs, into which additional elements, such as an inducible caspase-9 gene (iCas9) that can lead to self-destruction by apoptosis, were integrated (Fig. 2a). Among the clinical trials using a second-generation CAR construct, 4-1BB is more prevalent in the CAR construct. A total of 101 clinical trials (64.7%) utilized the 4-1BB costimulatory molecule, and 30 trials used CAR constructs with the CD28 intracellular signaling domain (19.2%). Among the clinical trials using third-generation CAR-T cells, approximately half of the trials (14) used the CD28-4-1BB CAR construct. Other constructs, including CD28-OX40, CD28-4-1BB-CD27, NKG2D-DAP10, CD28-TLR2, or iMyD88/CD40, were used in the CAR-T cells (Fig. 2b).

Among CAR-T cells using these different intracellular constructs, a comparison of CAR-T cells using CD28 or 4-1BB as the costimulatory molecule has been made, showing that CD28 and 4-1BB CAR-T cells vary in function and effectiveness^261,270,272,282–286^. CD28ζ and 4-1BBζ CARs show increased secretion of cytokines, including IFN-γ, IL-2, TNF-α, and GM-CSF, compared to first-generation CARs^150,276,277,287–290^. CAR T cells incorporating the 4-1BB costimulatory domain are more persistent and are likely to be more resistant to exhaustion than those incorporating CD28^261,282^. The incorporation of 4-1BB in the CAR construct may endow T cells with long-lasting survival and more central memory compartments than that of CD28^286,291,292^. 4-1BBζ CARs affect downstream signaling pathways through TNF receptor-associated factor 2 (TRAF2), while CD28ζ CARs affect signaling through nuclear factor (NF)-κB, AKT, extracellular signal–regulated kinase, NF of activated T cells, and Bcl-X_L_^260,293–298^. In clinical trials, CD28ζ CAR and 4-1BBζ CAR-T cells showed similar antitumor activities and remission rates^27,275^. Preclinical studies have shown that third-generation CAR-T cells exhibit improved antitumor effects compared with second-generation CAR-T cells^299,300^. Ramos et al.^301^ designed a clinical trial in which second- (CD28ζ) and third-generation (28BBζ) CD19 CAR-T cells were simultaneously infused into patients, and the third-generation CAR-T cells showed superior expansion and longer persistence than the second-generation CAR-T cells, suggesting the improved antitumor efficacy of the third-generation CAR-T cells compared to the second-generation CAR-T cells. Haso et al.^302^ reported that second-generation CAR-T cell immunotherapy was superior to third-generation CAR-T therapy with an anti-CD22 CAR in B-ALL, in contrast to the results of Ramos et al.’s research^301^. In fact, CAR functionality is not solely determined by the cytoplasmic signal domains, as other structural features may affect its overall function. CAR function varies depending on the expression level of the antigen on the target cells and the CAR in the T cells, the affinity of the CAR for the antigen, and signal pathway transduction by the CAR. To further determine the optimal choice of CAR-T for cancer patients, more clinical evidence is needed. For instance, a clinical trial (NCT01853631) is underway to compare the use of second- and third-generation CD19 CAR-T cells for the treatment of hematological malignancies, which will provide particular value for selection of second- and third-generation CAR-T cells.

CAR-T cells bearing costimulatory molecules other than CD28 or 4-1BB have been heavily investigated to determine their advantages, and some of these CAR-T cells are being tested in clinical trials for the treatment of cancers. CAR-T cells targeting CD33 with OX40 or CD28 costimulatory intracellular domains have shown similar effects to those with the 4-1BBζ construct in terms of T cell proliferation and cytokine secretion^303^. Third-generation GD2-specific CAR-T cells with OX40 and CD28 intracellular costimulatory domains were assessed (NCT02107963, NCT01822652, NCT01953900). ICOSζ CAR-T cells have increased phosphoinositide-3 kinase activation and IFN-γ production and secrete increased amounts of IL-17A, IL-17F, and IL-22 compared to 4-1BBζ CAR-T cells and express CD161, but their antitumor effects were not greater than those of CD28ζ and 4-1BBζ^303–305^ cells. CAR-T cells using a third-generation CAR construct containing inducible T cell co-stimulator and a 4-1BB intracellular costimulatory domain were demonstrated to have robust antitumor efficacy in solid tumor models compared with second-generation CAR-T cells having a 4-1BB domain^275,306,307^. Adding CD27 to the second-generation CAR construct increased T cell expansion, BCL-X_L_ upregulation, and IFN-γ production, and the resulting cells showed comparable in vivo efficacy compared with CD28ζ and 4-1BBζ CAR-T cells^308^. In addition, clinical data demonstrated that CD28-CD27-CD3ζ CAR-T cells were safe when infused to treat leukemia^262,308^. The intracellular domain of NKG2D, either individually or in combination with CD28/4-1BB domains, enhanced many properties of CAR-T cells, including the secretion of IFN-γ, TNF-α, and GM-CSF, and improved antitumor efficacy in vivo^309–313^. CAR-T cells containing a MyD88/CD40-based inducible costimulatory molecule promoted T cell proliferation and cytokine production^267^. A clinical trial (NCT02744287) exploited MyD88/CD40 CAR-T cells to target PSCA for the treatment of prostate adenocarcinoma and other cancers. Toll-like receptors (TLRs) and cytokine receptors can stimulate T cells to proliferate and produce inflammatory cytokines. The TLR2 and IL15Rα intracellular domains were also incorporated into the CAR construct, and the CAR-T cells were able to induce T cell expansion, cytokine production, and antitumor activity in vivo^266,268^. A CAR-T cell bearing the TLR-2 costimulatory domain was tested in a clinical trial to treat leukemia (NCT02822326). It is well accepted that second- or third-generation CAR constructs containing CD28 or 4-1BB intracellular costimulatory domains improved the persistence and survival of CAR-T cells. To overcome the insufficient persistence of TCR-T cells^314^, the addition of the CD28 or 4-1BB intracellular region was used to improve the tumor-killing capacity and long-term survival^315^.

Similar to CARs in CAR-T cells, the structures of TCRs in TCR-T cells have been optimized. TCR-T cells recognize tumor antigens depending on the HLA molecule used^316,317^. TCRs, which are composed of transgenic α chains and β chains, must bind to CD3 molecules. In genetically modified TCR-T cells, transgenic α or β chains can be mismatched with endogenous TCR α or β chains^318,319^. To minimize these mismatches, investigators introduced cysteine residues into the C-terminal domains of α and β chains and utilized the disulfide bond that formed between the cysteine residues to facilitate the assembly of the transgenic TCR α and β chains^320,321^. Other strategies have also been employed to prevent mispairing, such as mutating the C-terminal domains of the transgenic α and β chain to promote correct binding^322^. In addition, endogenous TCRs can be knocked out, which eliminates issues caused by TCR mispairing^323,324^. Indeed, endogenous TCRs also interfere with the function of CAR-T cells. When encountering p-HLA, the downstream signaling pathway initiated by CAR binding with the antigen was inhibited^325^. Thus endeavors have been made to replace the endogenous *TCR* gene with the *CAR* gene by a gene-editing tool to eliminate the interference due to TCRs, ensure uniform CAR expression, and increase antitumor potency^68,326^. In addition to removing TCRs, using a gene-editing tool to remove molecules governing immunological rejection, such as the β-2 microglobulin of HLA and other immune resistance factors, generated universal CAR-T cells with promising capacity for the treatment of hematological and solid tumors^327–329^, but more clinical evidence is needed to demonstrate the safety and efficacy of universal CAR-T cells^330^.

### Infusion strategy

The infusion strategy is important for the efficacy and safety of genetically engineered T cells. The intravenous infusion of CD19 CAR-T cell therapy has achieved success in targeting CD19+ B cell lymphoma^24,77,83^, and other CAR-T cells have targeted CD20, CD30, CD33, or BCMA for the treatment of hematological malignancies via intravenous infusion^23^. CAR-T cells were also intravenously infused in pilot trials for treating solid tumors. Their efficacy was restricted in the treatment of solid tumors^331^; it was found that intravenously infused CAR-T cells accumulated in the lung, liver, and kidney^29^, and only a small proportion of the infused cells homed in to the tumor site. The local delivery of CAR-T cells is thus encouraging. Multiple intracranial infusions of IL13Rα CAR-T cells, by enhancing infiltration and persistence, resulted in glioblastoma regression^31^. Recently, Smith et al. produced a novel cell biopolymer device, in which CAR-T cells proliferated robustly, and transplanted the device with CAR-T cells directly into tumor tissue, significantly improving CAR-T cell trafficking and infiltration^332^.

## Other challenges for engineered T therapy

Genetically engineered T cells have benefited patients in the treatment of tumors, but other obstacles beyond the issues discussed above have challenged the application of genetically modified T cells, even in the treatment of hematological malignancies^28,77,81,213,271,333–337^.

### Failure of CAR-T cell generation

Although the total number of CAR-T cells used for infusion is small (10^8^–10^9^), the failure to produce sufficient engineered T cells has been observed, which challenges the pharmaceutics of this therapy. Many factors can result in the failure of CAR-T production. Cancer patients generally undergo chemotherapy and other regimens, leading to lymphopenia, which affects the quality and quantity of harvested T cells. Leukapheresis improves the quality and purity of T cells, but poor expansion of CAR-T cells after engineering impedes the generation of sufficient numbers of modified T cells. T cells with different phenotypes exert distinct functions. Previous studies demonstrated that CAR-T cells with a central memory T cell phenotype (CD8+CD45RA−CD45RO+CCR7+) are closely related to antitumor activity^225,338–340^. In addition to the central memory phenotype, the CD4+/CD8+ ratio and the differential expression of other molecules in the infused T cells might affect the antitumor capability of CAR-T therapy^341,342^. Therefore, obtaining enough CAR-T cells for cancer immunotherapy means not only generating a sufficient number of T cells but also producing a defined phenotype and a specified ratio of subsets within the modified T cells.

There are many cell culture strategies used for the production of CAR-T cells. Magnetic beads coated with CD3/CD28 antibodies are often combined with cytokines, such as IL-2, IL-7, and IL-15, for the activation and expansion of CAR-T cells^225,343,344^. Artificial APCs are also used to greatly expand and generate antigen-specific CAR-T cells^345^. CD4- and CD8-positive cells can be enriched for producing CAR-T cells, and a defined ratio of CD4 and CD8 cells can ultimately be ensured^341^. In addition to differences in the expansion protocol, CAR-T cells with different intracellular costimulatory domains in their CAR constructs exhibited different expansion and phenotype properties^263,286,304,307,308,346,347^. The lengths of the CAR extracellular spacers used are critical to CAR-T cell therapy^348^. It is likely that formulating CAR-T cells with a defined composition of different subsets can enhance the uniformity of CAR-T products, which could be used in further investigations to draw more definitive conclusions.

### Relapse after CAR-T therapy

Although CD19-targeted CAR-T cells achieved dramatic antitumor efficacy in treating hematological malignancies^27,275,341,349,350^, a considerable number of patients with CR have relapsed after CAR-T therapy. Although the mechanism of relapse has not been fully elucidated, antigen loss and poor CAR-T persistence are thought to contribute to the relapse of disease.

Tumors in a small proportion of pediatric and adult responders relapsed after treatment with CD19 CAR-T cells because of CD19 deletion and CD19 tumor cell growth^81,351,352^, which results from acquired resistance to CAR-T therapy due to antigen escape^90,353,354^. To prevent the recurrence of the tumor, broader immune activation is required to elicit a second wave of immunity in addition to CAR-T cell therapy^355^. Recently, other novel mechanisms accounting for the relapse of hematological malignancies after CD19 CAR-T cell therapy have been reported. CD19 molecules on tumor cells transduced with CAR vectors can be masked by CAR molecules, leading to their escape from attack by CAR-T cells and the recurrence of malignancy^88^. A target antigen recognized by CAR-T cells on the tumor cells can even be removed by CAR-T cells through trogocytosis, resulting in a reduction in the antigen and fratricide between CAR-T cells^89^. Another obstacle is the poor persistence of CAR-T cells. The efficacy of CAR-T cell therapy is closely related to the in vivo expansion and survival of cells^356^. Many strategies discussed above, including the optimization of the CAR construct and preconditioning and infusion strategies, can prolong the survival of CAR-T cells and enhance their antitumor efficacy.

### Side effects

CAR-T cells target the antigen expressed on the surface of tumor cells. This antigen is generally expressed on normal cells and not exclusively on tumor cells. CAR-T cells attack normal tissues when clearing tumor cells, resulting in side effects in normal tissues, which are called “on-target, off-tumor” side effects. Clinical studies in which CAR-T cells infused for cancer therapy have recognized HER2^188^, CAIX^357^, CD19^358,359^, and mesothelin^360^ have shown damage to normal tissues. Many strategies have been developed for enhancing the safety of CAR-T cell therapy, such as the targeting of other antigens, such as CD1a, in place of CD19^361^, equipping CAR-T cells with safety switches by introducing suicide genes^362–365^, expressing a split CAR molecule^366^, constructing inhibitory CAR constructs^367^ or dual antigen-activated CAR-T cells to recognize tumor cells more specifically^368^, and tuning the affinity of the scFv used for the CAR construction^244,245,369^ to minimize the incidence of severe adverse effects. In addition, the local injection of CAR-T cells can also reduce off-target effects, since the CAR-T cells are regionally restricted^212^. Further research may seek to improve the safety of CAR-T cells, especially regarding the selection of the CAR-recognized antigen, the development of controllable CAR-T cells, and the optimization of infusion strategies.

Among the side effects of CAR-T cells, CRS is the most common adverse effect. CRS resulting from CD19 CAR-T cell therapy is tolerable if properly treated^26,27,83,370^. CRS is induced by the robust activation of the immune system during CAR-T cell therapy, triggering the release of a large number of cytokines, including IL-6, IL-10, IFN-y, TNF-α, GM-CSF, and other cytokines, and causing severe adverse effects^371,372^. The severity of the CRS-related side effects is closely related to the amounts of released cytokines^373^, tumor burden^374,375^, and the structure of the CAR^272^. mAbs against IL-6Ra (tocilizumab or sarilumab) are used for the treatment of CRS in addition to the use of corticosteroids to inhibit inflammatory reactions^152^. The blockade of IL-1β^376^ or TNF-α^152^ is another strategy used to treat CRS in addition to IL-6, but the benefits for patients are yet to be documented. GM-CSF-knockout CAR-T cells resulted in minimal incidence of CRS compared with GM-CSF-intact CAR-T cells^372^, indicating the pivotal role of CAR-T-derived GM-CSF in the occurrence of CRS.

In addition to CRS, varying degrees of neurotoxicity have occurred as a result of the clinical application of CAR-T cell therapy for treating hematological tumors^377–379^. Indeed, neurotoxicity is always closely related to CRS. Mild neurotoxicity is transient and reversible. The appearance of acute and severe neurotoxicity is usually accompanied by severe CRS symptoms due to the increased permeability of the blood–brain barrier. Although the mechanism of the incidence of neurotoxicity is largely unknown, many clues have indicated that inflammatory cytokines affect the function of the blood–brain barrier and endothelial cells, contributing to neurotoxicity^380,381^. In nonhuman primates, CAR-T cell-mediated neurotoxicity was also confirmed to be associated with pro-inflammatory CSF cytokines and pan-T-cell encephalitis^382^. For the treatment of neurotoxicity caused by CAR-T cell therapy, a regimen should be prescribed that is dependent on the severity. Anti-IL-6 therapy can reverse side effects at the early stage. For the treatment of late-stage neurotoxicity, corticosteroids are recommended. Patients with nonconvulsive and convulsive status epilepticus should be managed with benzodiazepines and additional antiepileptics^383,384^.

TCR-T cells target HLA-presented antigens on tumor cells. The potential side effects of TCR-T cell therapy are decreased compared to those of CAR-T cell therapy^385^. However, safety concerns should be paid more attention when the affinity or other characteristics of the TCR are modified. TCR-T cells with enhanced affinity always improve the recognition of the HLA-presented antigens, but side effects usually accompany this. In a pilot study using affinity-enhanced TCR-T therapy for the treatment of melanoma and other cancers, two of the nine patients died of leukoencephalopathy after treatment with MAGE-3 high-affinity TCR-T cells because of off-target toxicity^386^. In another clinical trial for the treatment of melanoma and myeloma, two patients died of cardiotoxicity after MAGE-3-specific, affinity-enhanced TCR-T cell therapy, mainly due to cross-reaction with the titin peptide, which is expressed in heart tissue^141^. Therefore, meticulous investigation of affinity-enhanced TCR-T therapy is needed before it can provide clinical benefit to cancer patients.

## Outlook

Among cancer immunotherapies, CAR-T cell therapy has shown robust antitumor efficacy for the treatment of hematological malignancies. Upon the approval of CAR-T cell therapy for the treatment of leukemia and lymphoma, more patients will be able to benefit from this new therapy, but side effects, relapses after CR, and long-term monitoring should be of concern. CAR-T cell therapy for solid tumors is far from satisfactory. Endeavors should be made to overcome immunosuppression mediated by the microenvironment of solid tumors. TCR-T cells and other genetically engineered immune cells have produced effective immunity during cancer treatment in preclinical and clinical trials, and efforts should be made to enroll more patients to obtain the benefits of TCR-T therapy. It is hoped that another breakthrough in gene-engineered-T cell therapy for tumors is coming soon.
